# Sensorimotor Modulation of Mood and Depression: In Search of an Optimal Mode of Stimulation

**DOI:** 10.3389/fnhum.2013.00428

**Published:** 2013-07-30

**Authors:** Resit Canbeyli

**Affiliations:** ^1^Psychobiology Laboratory, Department of Psychology, Bogazici University, Istanbul, Turkey

**Keywords:** depression, amygdala, mood regulation, sensorimotor activation, physical exercise, affective neurocircuitry

## Abstract

Depression involves a dysfunction in an affective fronto-limbic circuitry including the prefrontal cortices, several limbic structures including the cingulate cortex, the amygdala, and the hippocampus as well as the basal ganglia. A major emphasis of research on the etiology and treatment of mood disorders has been to assess the impact of centrally generated (top-down) processes impacting the affective fronto-limbic circuitry. The present review shows that peripheral (bottom-up) unipolar stimulation via the visual and the auditory modalities as well as by physical exercise modulates mood and depressive symptoms in humans and animals and activates the same central affective neurocircuitry involved in depression. It is proposed that the amygdala serves as a gateway by articulating the mood regulatory sensorimotor stimulation with the central affective circuitry by emotionally labeling and mediating the storage of such emotional events in long-term memory. Since both amelioration and aggravation of mood is shown to be possible by unipolar stimulation, the review suggests that a psychophysical assessment of mood modulation by multimodal stimulation may uncover mood ameliorative synergisms and serve as adjunctive treatment for depression. Thus, the integrative review not only emphasizes the relevance of investigating the optimal levels of mood regulatory sensorimotor stimulation, but also provides a conceptual springboard for related future research.

## Introduction

Major depression, second only to hypertension as the most frequent illness in the world and projected to be the leading disease burden by 2020 (Lopez and Murray, 1998) has debilitating personal and socioeconomic consequences. Despite a long history of clinical investigations and basic research, the neurobiological basis of mood disorders is still not fully understood and successful outcome of treatment in the form of psychotherapy and/or pharmacotherapeutics is far from certain (Rush and Thase, 1977; Kessler et al., 2003; Trivedi et al., 2006b).

Research on the etiology and treatment of depression has focused on complex affective and cognitive factors that figure in mood disorders. This “top-down” approach is supported by monoaminergic pharmacotherapy of depression based on improving a dysfunctional central mood neurocircuitry comprising the frontal cortices, the cingulate gyrus, the amygdala, the hippocampus, hypothalamus, and the basal ganglia (Ongur et al., 1998; Heimer, 2003; Heimer and Van Hoesen, 2006; Drevets et al., 2008; Koenigs and Grafman, 2009; Price and Drevets, 2010; Pandya et al., 2012). While this approach has produced new insights into and therapeutic tools for mood disorders, a recent integrative review called attention to the fact that a diametrically opposite perspective, namely a “bottom-up” approach, that begins with peripheral sensorimotor stimulation can also be informative in understanding the factors that modulate mood, precipitate depression and also provide new insights into therapeutic techniques (Canbeyli, 2010). Briefly, the review presented evidence that sensorimotor stimulation through unimodal sensory or motor channels is capable of modulating mood and depressive symptoms. Moreover, it was shown that the peripheral stimulation impacted the constituents of the affective fronto-limbic neurocircuitry implicated in mood regulation and depression by studies based on the top-down approach. The present paper aims to expound on the bottom-up approach for several reasons. First, it will be shown selectively in the domains of vision, audition, and physical exercise that sensorimotor stimulation is capable of both improving and aggravating mood and depressive symptoms and does so by activating the same mood neurocircuitry implicated in depression. Second, it will be argued that the amygdala plays a pivotal role in the reception, emotional evaluation, and storage of the mood regulatory sensorimotor stimulation. Finally, it will be proposed that a methodical investigation into the combination of multimodal sensorimotor stimulation can provide new insights into mood regulation and for new therapeutic techniques. The overarching aim of the present paper is to draw attention to the possibility that there may be an optimal level of sensorimotor stimulation for maintenance of a positive mood. As will be shown below, excessive or deficient unimodal sensorimotor stimulation can lead to dysphoric mood and possibly depression. On the other hand, sensorimotor stimulation can also have positive effect on mood depending on the quality, intensity, and duration of stimulation. It is therefore important to undertake parametric and systematic investigation of the impact of combinations of sensorimotor stimulation to explore the potential of an optimal psychophysical level of stimulation for psychological well-being.

## Sensorimotor Modulation of Mood and Mood Disorders

In real life, humans and animals face a stream of sensorimotor stimulation with considerable qualitative and quantitative variations. This bombardment does not normally result in any lasting psychological ill effects. However, it is a fact of life that continuous stress and excessive stimulation can have transient and possibly lasting psychopathological effects. In fact, prolonged stress is considered a major source of psychopathology both in humans and animals (Tafet and Bernardini, 2003; McEwen, 2005). Psychopathology inducing stress is often a complex array of events and sensorimotor activation that does not lend itself to a precise analysis of the critical detrimental factors. Similarly, in treatment of mood disorders in humans and in animal models, it is difficult to assess the ameliorative impact of components of therapy or antidepressant treatment. In contrast, the present section provides evidence for the visual and auditory modalities and physical exercise that unipolar sensorimotor activation is capable of modulating mood and depressive symptoms.

### Light modulates mood

The mood modulatory nature of visual input is shown by the fact that photic stimulation can alleviate, whereas impaired vision or inadequate light reception can aggravate depressive symptoms (Espiritu et al., 1994; Jean-Louis et al., 2005).

#### Light exposure improves mood and alleviates depressive symptoms

Exposure to artificial bright light in the morning or early evening successfully alleviates symptoms of both seasonal (Goodwin et al., 1982; Lewy et al., 1982, 1987; Wehr and Wirz-Justice, 1982; Rosenthal et al., 1984; Rosenthal and Wehr, 1992; Oren and Terman, 1998) and non-seasonal depression (Mackert et al., 1991; Even et al., 2008). Similar ameliorative effects of light exposure have been reported in non-clinical populations. Thus, exposure to artificial bright light ameliorates mood in subjects with or without winter-related mood symptoms (Partonen and Lönnqvist, 2000). Bright-light exposure for 8 weeks paired with exercise significantly reduces depressive symptoms in non-clinical subjects compared to exercise alone under normal ambient illumination (Leppamaki et al., 2002).

Several studies indicate that prolonged exposure to light by means of long photoperiods has ameliorative effect on depressive symptoms in animals. A month-long exposure of male rats to a long photoperiod (15 h L:9 h D instead of the usual 12 h L:12 h D daily light/dark cycle) has an antidepressant effect (Molina-Hernandez and Tellez-Alcantara, 2000). There are also reports that a much shorter exposure to light exposure can have antidepressant effect in rats. Three studies using behavioral despair model of depression indicated antidepressant effect of photic stimulation in the dark phase of an 12 h L/12 h D cycle when rats were exposed to a light pulse of 12-h (Yilmaz et al., 2004), 30-min (Schulz et al., 2008), or even 10 min duration (Iyilikci et al., 2009). Quality of photic stimulation can also be a factor in modulating mood. Clinical practice (Brainard et al., 1990; Glickman et al., 2006) as well as animal research (Iyilikci et al., 2009) indicates that the wavelength of light exposure may also be critical in that blue but not red light is effective in treating depression in humans and in providing antidepressant effect in forced swimming in rats.

#### Inadequate or inappropriate light exposure aggravates depressive symptoms

Reduced illumination due to short days in the winter is a globally experienced source of lowered mood and exacerbated depressive symptoms as in the case of Seasonal Affective Disorder (SAD; Lewy et al., 1982, 1987; Wehr and Wirz-Justice, 1982). Depression is more likely in areas with poor sunlight exposure throughout the year (Booker et al., 1991). On an individual basis, patients with compromised photoreception due to ophthalmic dysfunctions show more depressive symptoms (Rovner and Shmuely-Dulitzki, 1997; Shmuely-Dulitzki and Rovner, 1997; Rovner et al., 2002).

Reduced light exposure due to artificially shortened photoperiods aggravates depressive symptoms in animals as well. Depression-like behavior was induced by short light periods in diurnal animals such as the sand rats (Einat et al., 2006) and the unstriped Nile grass rat (Ashkenazy-Frolinger et al., 2010) and by prolonged total light deprivation in the Mongolian gerbil (Lau et al., 2011). Importantly, depression induced by a 3-week short day in sand rats was alleviated by daily bright-light exposure (Ashkenazy et al., 2009). Depression-like behavior has been observed in hamsters exposed to short photoperiods in the nocturnal Siberian hamsters (Prendergast and Nelson, 2005; Pyter and Nelson, 2006; Workman et al., 2011). There is also indication that chronic light exposure in the dark phase an L:D cycle can have depressogenic effect. Dim light exposure in the dark phase of an L/D cycle induced depressive behavior in female mice compared to controls exposed to total darkness in the dark phase of the L/D cycle (Bedrosian et al., 2011). Similarly, mice exposed to constant light throughout the day (LL schedule) displayed sucrose anhedonia and depression-like behavior in a forced swim test (Fonken et al., 2009). A recent study indicated that depression-like behavior was induced by an aberrant light schedule (3.5 h L:3.5 h D cycle) that did not disrupt the circadian rhythms of mice (LeGates et al., 2012).

### Auditory stimulation modulates mood and depressive symptoms

For humans, auditory stimulation is an effective means of regulating emotion and mood. Moreover, humans are more likely to assess emotions by a combination of visual feedback and auditory perception than either alone (Bouhuys et al., 1995; de Gelder et al., 1999a,b).

#### Auditory stimulation can improve mood and depressive symptoms

A variety of auditory stimulation ranging from musical compositions to noise can modulate mood. Listening to different types of music can alleviate depressive or anxious mood (Clark, 1983; Pignatiello et al., 1986; Albersnagel, 1988; Bouhuys et al., 1995; Smith and Noon, 1998; Gagner-Tjellesen et al., 2001; Thompson et al., 2001; Tornek et al., 2003; Hsu and Lai, 2004; Gold et al., 2009). Results of two studies attest to the wide range of auditory stimulation that can affect mood. Lane et al. (1998) reported that binaural auditory beats induced by presenting two tones differing slightly in frequency to the two ears modulate mood and psychomotor performance. Exposure to musically enhanced birdsong early in the morning induces positive mood and lowers depressive symptoms (Goel and Etwaroo, 2006).

#### Auditory stimulation or hearing impairment can aggravate depressive symptoms

Listening to music can induce depressive or anxious mood depending on the nature of the composition (Pignatiello et al., 1986; Smith and Noon, 1998; Thompson et al., 2001; Abbott, 2002; Kemper and Danhauer, 2005). Support for the mood regulatory effect of auditory stimulation is provided by several studies that show a positive correlation between problems of hearing and depressive symptoms. Depression is more prevalent in patients with inner ear disorders than in healthy controls (Berrios et al., 1988) and, in general, an increased rate of depressive symptoms is observed in people with hearing impairment or total deafness (Magilvy, 1985; Leigh et al., 1989; Watt and Davis, 1991; Steinberg et al., 1998; Leigh and Anthony-Tolbert, 2001; Zazove et al., 2006). Furthermore, tinnitus, a hearing disorder, is significantly correlated with depression (Stephens and Halam, 1985; Sullivan et al., 1988; Budd and Pugh, 1995; Langguth et al., 2011).

Corroborating findings with humans, hearing deprivation has been shown to have depressive effect in rats. Temporary induction of deafness in rats results in depression as measured by increased immobility in forced swim tests; the negative effect was abolished when hearing was restored (Drago et al., 1996). A few studies using animal models of depression report depressant effects of auditory stimulation. Exposing rats to white noise in a novel environment for 30 min significantly reduced struggling behavior in a swim test compared to controls (Weiss et al., 2000). Similarly, high intensity sinusoidal tone stimulation induced depression-like symptoms in rats in forced swim tests (Bulduk and Canbeyli, 2004). More recently, a study indicated that exposing rats to daily loud noise for 15 days resulted in anxiety and depression-like behavior as measured by tail suspension test (Naqvi et al., 2012).

### Physical exercise modulates mood and depressive symptoms

The motor system has a dual relationship with the central mood regulatory network; it not only serves as the substrate for psychomotor symptoms of depression, but can also affect the circuitry that is responsible for mood by means of its direct and indirect bidirectional connectivity with the frontal cortices and the amygdala (Marchand and Yurgelun-Todd, 2010, 2012; Marchand et al., 2012). Moreover, physical activity can indirectly contribute to regulation of mood by providing multisensory stimulation arising out of ambulation. Psychomotor disturbances in depression either as an increase in activity or more frequently as psychomotor retardation are recognized by the DSM-IV classification as primary feature of depressive disorders. Stress, negative mood, and depression can alter psychomotor activity, usually in the direction of reduced mobility in humans (Weiss et al., 1974; Kupfer et al., 1975; Szabadi et al., 1976; Farmer et al., 1988; Dunn et al., 2001) and animals (Walsh and Cummins, 1976; Katz et al., 1981). Conversely, emotions can modulate postural adjustments (Hillman et al., 2004; Horslen and Carpenter, 2011), gait (Naugle et al., 2011), and physical effort (Schmidt et al., 2009).

#### Exercise improves mood and alleviates depression

Amelioration of depressive symptoms have been reported by several weeks of physical activity such as running or outdoor walking (Morris et al., 1990), aerobics (Martinsen et al., 1985; Freemont and Craighead, 1987; Dimeo et al., 2001), and weight training (Doyne et al., 1983). Animal studies provide further support for the ameliorative effect of physical exercise on depressive symptoms induced by stressful situations (Dey, 1994; Solberg et al., 1999; Greenwood et al., 2003; Hoomisen et al., 2003; Bjornebekk et al., 2005; Zheng et al., 2006; Duman et al., 2008; Greenwood and Fleshner, 2008).

Ameliorative effect of exercise in depressed patients was found to be comparable to psychotherapy or treatment with medication observable even in a 1-year follow-up evaluation (Klein et al., 1985). A 4-month regimen of aerobic exercise by MDD patients was as effective in improving depressive symptoms as sertraline treatment, resulting in lower rates of relapse in a 10-month follow-up study (Babyak et al., 2000). Similarly, a 4-month-long exercise program for patients with major depression resulted in remission rates comparable to a group receiving sertraline and higher than that of an untreated placebo group (Blumenthal et al., 2007).

#### Lack of exercise or exercise withdrawal induces depressive symptoms

An inverse relationship exists between activity and depression such that exercise even in low doses or for short durations reduces likelihood of depression (Teychenne et al., 2008; Helmich et al., 2010) whereas lack of activity constitutes a risk factor for depressive symptoms (Farmer et al., 1988). Moreover, individuals with sedentary life styles display more depressive symptoms than those who regularly engage in physical activity (Dunn et al., 2001, 2005; Goodwin, 2003) and decreased physical activity over the years increases the likelihood of depressive symptoms among the elderly (Lampinen et al., 2000; Rovner et al., 2002). In general, a decrease in general level of physical activity over time is a risk factor for depression, while increasing activity over the years protects against depression (Farmer et al., 1988; Goodwin, 2003).

Results of experimentally controlled withdrawal of exercise in healthy people who regularly exercise also point to the negative effect of reduced physical activity on mood and depressive symptoms. For instance, negative mood and depressive symptoms emerge in the first week of exercise withdrawal in individuals asked to forego their regular exercise regimen for 2 weeks (Berlin et al., 2006). Similarly, abstaining from running in the middle 2 weeks of a 6-week exercise in healthy individuals results in more symptoms of depression in both weeks compared to those allowed to run throughout the 6-week period. Importantly, returning to regular running alleviated the negative effects in the last 2 weeks of the study (Morris et al., 1990).

## Neuroanatomical Basis of Sensorimotor Stimulation

Evidence garnered from several lines of research including neuroimaging studies, deep brain stimulation, and research with animal models indicate that mood disorders including depression are not due to a focal pathology but arise out of dysfunction in an affective neurocircuitry consisting of several cortical and subcortical structures. The main constituents of this fronto-limbic neurocircuitry are the ventromedial and dorsal prefrontal cortices (VMPFC and DPFC), anterior cingulate cortex (ACC), amygdala, hypothalamus, hippocampus, nucleus accumbens, and the basal ganglia (Mayberg, 1997; Soares and Mann, 1997; Ongur et al., 1998; Tekin and Cummings, 2002; Heimer, 2003; Phillips et al., 2003a,b; Heimer and Van Hoesen, 2006; Drevets et al., 2008; Koenigs and Grafman, 2009; Lorenzetti et al., 2009; Preskorn and Drevets, 2009).

### Vision

Visual inputs can modulate mood by a cascade of processing that starts with the visual cortices, continuing with the ventral stream that projects to the fronto-limbic structures, namely the orbitofrontal cortex, the ventromedial prefrontal cortex, the cingulate gyrus, the hippocampus, and the amygdala. Additionally, light exposure can modulate mood via the suprachiasmatic nucleus (SCN), the master oscillator in the basal hypothalamus. SCN has connections with constituents of the fronto-limbic circuitry involved in mood regulation such as the amygdala, the bed nucleus of the stria terminalis, shown to be involved in depression in rats (Schulz and Canbeyli, 2001; Pezuk et al., 2006, 2008), the paraventricular nuclei of the hypothalamus and the thalamus as well as with the frontal cortical areas (Watts and Swanson, 1987; Watts et al., 1987; Vrang et al., 1995). Potential involvement of the SCN in affective behavior is shown by reports that bilateral electrolytic lesions of the SCN in rats (Tataroglu et al., 2004) and chemical lesions in mice (Li et al., 2009) prevent depression.

### Audition

Audiogenic stress affects the hypothalamic-pituitary-adrenal (HPA) axis (Irwin et al., 1989; Burrow et al., 2005; Midzyanovskaya et al., 2006) and auditory stimulation activates subcortical structures involved in mood, particularly the amygdala and the hippocampus (Singh et al., 1990a,b; Britton et al., 1992; Nahm et al., 1993; Romanski and LeDoux, 1993; Day et al., 2005; Mitterschiffthaler et al., 2007). Human neuroimaging studies indicate that pleasant music activates amygdala and the ventral striatum as well as the orbitofrontal and ventral medial prefrontal cortices (MPF) (Blood and Zatorre, 2001).

### Physical exercise

The basal ganglia and the thalamus form widespread connections with the ventromedial, orbitofrontal, and dorsolateral frontal cortices and the cingulate cortex by means of parallel but interacting circuits (the basal ganglia/thalamocortical circuits; Alexander and Crutcher, 1990). Subcortically, the basal ganglia and thalamus interact with several mood regulatory limbic structures, including the nucleus accumbens, ventral striatum, amygdala, raphe nuclei, and hippocampus (Powell and Lehman, 1976; Russchen and Price, 1985; Berchtold et al., 2001, 2002; Heimer, 2003; Heimer and Van Hoesen, 2006). Involvement of the basal ganglia in depression is indicated by decreased basal rCBF (Curran et al., 1993) and volumetric reductions (Husain et al., 1991; Soares and Mann, 1997; Lorenzetti et al., 2009) and dysfunctional cortico-basal connectivity (Marchand et al., 2012) in MDD patients.

## Amygdalar Involvement in Sensorimotor Modulation of Mood

The evidence presented above indicates that a diverse range of sensory or motor inputs (or their lack) can modulate mood and depressive symptoms. Moreover, there is a wide temporal span in which the affective impact of such input can be achieved, ranging from a few minutes to days and beyond. It is therefore important to elucidate the process by which the initial reception of sensorimotor input by peripheral channels is converted to a more or less stable state that can be characterized as amelioration or exacerbation of mood and depressive symptoms.

Living organisms are almost constantly bombarded with sensory input in the waking state and often engage in physical activities without obvious affective residues. The sequence of events that connect sensorimotor stimulation with the central mechanisms of mood regulation therefore must follow a triadic process of initial reception of stimulation by central structures, followed by the steps of emotional labeling and recording into long-term memory of those inputs that matter for the organism. Clinical observations, research with animals, and particularly accumulation of neuroimaging data have all contributed to the view that there is no structure pathognomonic to depression. It is therefore not possible to assign any of the phases in the triadic process to a single brain structure. Moreover, neuroanatomical evidence cited previously indicates that several structures forming a widespread fronto-limbic network are affected by different types of mood regulator sensorimotor stimulation. Nevertheless, the present paper will argue that the amygdala (or rather the amygdalar complex) plays a pivotal role (arguably as “*primus inter pares*”) in all three phases of the triadic process in converting the centrally received sensorimotor stimulation to mood regulatory processes.

### The amygdala is a gateway for mood regulatory sensorimotor stimulation

A large number of studies on the neuroanatomy and functions of the amygdala has provided a complex picture of amygdalar connectivity and involvement in diverse phenomena such as sensory evaluation, learning, memory, and mood regulation (LeDoux, 2007; Pessoa, 2011). Briefly, the amygdala consists of several nuclei which differ among themselves in terms of neuroanatomical and neurochemical properties as well as intra- and extra-amygdalar connectivity. The major nuclei involved in the functions related to processing sensory information and affective function are the basolateral nucleus (BLA; consisting of the lateral, basal, and accessory basal nuclei), the medial nucleus and the central nucleus.

The BLA is the recipient of input from multiple sensory systems both from the thalamus and cortical areas, while the medial nucleus receives projections from the olfactory bulbs. The central nucleus receives intra-amygdalar inputs from the lateral and basal nuclei directly or indirectly via the inhibitory intercalated cells and extra-amygdalar inputs from the viscero-sensory information from the cortex and the brainstem. The central nucleus constitutes the major output channel of the amygdala modulating several visceromotor functions by means of its connections with the hypothalamus and lower brainstem structures. The central nucleus also contributes to the regulation of corticosteroid hormone release in response to arousal or stress via the HPA axis.

### The amygdala is involved in emotional assessment (“labeling”) of mood regulatory sensorimotor stimulation

A major function of the multifarious amygdala is emotional assessment. The well established amygdalar involvement in fear conditioning (see Phelps and LeDoux, 2005) may have initially biased thinking about amygdalar involvement in emotional evaluation of sensory input. The amygdala was thought to be responsive mainly to negative (or aversive) stimulation and not specially reactive to positive stimuli. There is now abundant evidence to show that the amygdala is activated by positive as well as negative stimulation (Rasia-Filho et al., 2000; Davis and Whalen, 2001; Garavan et al., 2001; Hamann et al., 2002).

There is however no consensus on the critical emotional aspect or dimension of sensory input that results in optimal activation of the amygdala. A major inspiration to define the nature of such stimulation is based on the circumplex model of emotion that characterizes emotion inducing stimulation in terms of two dimensions: valence and arousal (Russell, 1980, 2003). Thus, amygdalar activation and assessment of positive or negative stimuli (or situations) has been considered in terms of valence (how positive or negative) or arousal (how exciting or calm) (Silke et al., 2008; Karlsson et al., 2010; Groenewold et al., 2013). While there is evidence that these dimensions can account for the emotional aspect of diverse types of stimulation impinging on an organism, recent studies, particularly utilizing neuroimaging techniques, have shown that the central reaction, including that of the amygdalar complex, to peripheral stimulation is not totally understandable in terms of just valence or arousal. Several studies have found that amygdalar response to such stimulation can be more appropriately characterized in terms of intensity (Surguladze et al., 2003; Anders et al., 2004), salience (Liberzon et al., 2003; Anderson, 2005), relevance (Sander et al., 2003; Zald, 2003), meaningfulness (Phan et al., 2002; Sergerie et al., 2008), or disambiguation of potentially significant stimulation (Whalen et al., 2001).

A complication in attempting to classify sensorimotor stimulation in terms of its emotional impact on the amygdala is based on the fact that many studies investigating the relative contributions of these dimension (valence vs. arousal, for example) have used extreme values and have usually ignored the mid-range of these dimensions. However, evidence presented in the present review suggests that stimulation arising from of a wide range of events including hearing birdsong to engaging in physical activity can induce emotions and regulate mood. For instance, presentation of an innocuous stimulation such as a sinusoidal tone that is unlikely to have a strong affective quality when presented singly nevertheless can induce depressive symptoms after several repetitions (Bulduk and Canbeyli, 2004). Moreover, characterization of amygdalar response to environmental events and the studies providing empirical support for such labeling have almost all come from observations where stimuli variously thought to be arousing, valenced, salient, threatening, etc. have been present. One of the main points of the present review is that mood can be modulated not just by presence of a sensorimotor stimulation but also by its reduction and even total absence. For instance, reduced or absent auditory stimulation, light exposure, or even exercise can all modulate mood and depressive symptoms.

As shown by evidence presented above, a major aspect of research on amygdalar response to events including unimodal stimulation is driven by the motivation to categorize the nature of amygdalar activation at the interface between sensation and affective arousal and labeling. The main thrust of the present review in this respect is to elucidate the amygdalar activation at the interface between arousal and potential affective memory, that is, to delineate the amygdalar contribution to the transfer of affectively labeled events into long-term memory. From this perspective, it may be more appropriate to embrace a more general concept, namely “emotional tag” to characterize the emotional labeling of potentially significant stimulation by the amygdala (Richter-Levin and Akirav, 2003; Bergado et al., 2011). Briefly, the concept is based on the notion that emotional “weight” of an event activates the amygdala such that tagging facilitates the consolidation of the event in long-term memory. With its wide scope this functional characterization also dovetails with the concept of “synaptic tagging” proposed to explain the memory consolidation in specific synapses in response to particular activation (Frey and Morris, 1997, 1998a,b).

### The amygdala is involved in storing emotional inputs in long-term memory

Evidence cited previously indicates that a wide range of sensorimotor stimulation, varying in quality and quantity as well as temporal span can modulate mood and depressive symptoms. Accomplishment of such mood regulatory function must entail not just labeling of sensorimotor input but also keeping tract of past stimulation and their consequence as well: for sensorimotor stimulation to have a lasting effect on mood, the emotionally labeled or tagged input must be encoded into long-term storage. There is abundant evidence that humans (Bradley et al., 1992; Gallagher and Chiba, 1996; Hamann et al., 1996) and animals (Cahill and McGaugh, 1990, 1998) remember emotionally arousing events better than neutral events. In this respect, the amygdala plays a central role in that even though it is not in general involved in all aspects of memory (McGaugh et al., 1996) and is not the seat of long-term memory (Izquierdo et al., 1997; Packard and Teather, 1998; Packard and Gabriele, 2009; McDonald et al., 2010), it mediates the storing of emotional events in long-term memory (see McIntyre et al., 2012 for a recent review).

Support for the mediational role of the amygdala in long-term storage of emotional events comes from several types of research with animals and observations with humans. Stimulation of the BLA in animals facilitates long-term potentiation (LTP) and hippocampal plasticity (Ikegaya et al., 1995, 1997; Frey et al., 2001) and enhances specific sensory memory representations in the cerebral cortex (Chavez et al., 2009, 2013). Findings of human brain imaging and memory studies provide additional evidence that the amygdala and medial temporal lobe interact to consolidate memories of emotionally arousing material. In a study with patients with amygdalar pathology, memory loss for emotional items was directly related to the extent of amygdalar damage (Richardson et al., 2004). Similarly, amygdalar lesions in humans impair the normally observed enhancement of memory for emotional as compared with neutral stimuli (Cahill et al., 1995; Adolphs et al., 1997; Phelps et al., 1997).

### The amygdala is a nexus for top-down and bottom-up processes that modulate mood

The amygdala is not only a core structure in the bottom-up processes of mood regulation, but also constitutes a nexus in the interaction of top-down cortical factors with the limbic structures involved in depression. In addition to the evidence presented in Section “Neuroanatomical Basis of Sensorimotor Stimulation” above, a multitude of studies provide support for amygdalar involvement in the cortical and cognitive factors involved in regulation of mood (Price and Drevets, 2010). The amygdala has reciprocal connections with the prefrontal affective network including the dorsolateral (DLPF) and MPF and the ACC including the subgenual ACC (sgACC) (Drevets et al., 2004). Amygdalar volume and metabolic changes as well as neuropathologies have been reported in depression along with similar alterations in frontal brain structures such as the prefrontal cortex and the ACC (Drevets, 2001; Sheline et al., 2001; Radley et al., 2006; Koolschijn et al., 2009; Jahn et al., 2010). Importantly, depression severity in MDD is positively correlated with activity in the amygdala, sgACC and ventromedial frontal polar cortex (Drevets and Price, 2005), and negatively correlated with that in the left VLPFC/lateral OFC (Drevets et al., 2004).

Cognitive explanations of depression including the role of rumination emphasize the top-down frontal cortical influences on mood regulation and maintenance of depressive symptoms (Cooney et al., 2010; Disner et al., 2011). Increased negative cognitive processing and ruminative thought are consistently related to depressive symptoms (Roberts et al., 1998; Spasojevic and Alloy, 2001; Mor and Winquist, 2002) and are associated with increased activity in the amygdala and reactivity in the sgACC (Siegle et al., 2006). In fact, rumination has been consistently correlated with structures involved in top-down modulation of mood such as the lateral prefrontal cortex, MFC and the ACC (Putnam and McSweeney, 2008; Denson et al., 2009; Johnson et al., 2009; Kross et al., 2009), and the amygdala (Siegle et al., 2002; Ray et al., 2005).

## Conclusion and Future Directions

A major aim of the present paper is to draw attention to the importance of a bottom-up approach to mood and mood disorders that begins with peripheral sensory or motor inputs. The main reason for advocating such an approach is based on the fact that affective neuroscience has often ignored the abundant literature that indicates an important modulatory role for unimodal peripheral inputs. A major outcome/corollary of this predominantly one-way approach has been to emphasize the role of negative inputs (often centrally generated by rumination and fixations) in both the etiology and treatment of depression. In a recent review of a top-down view of mood disorders, cognitive model of depression, major elements of the model are biased attention, biased processing, biased thought and rumination, biased memory, and dysfunctional attitudes and schemas (Disner et al., 2011). There is of course a great deal of justification for dwelling on biases and negativity with respect to human depression. However, it is a fact that depression and mood disorders are common in many species and have served as useful models for humans (see Willner, 1995). It is also a fact that negativity has been overemphasized, particularly with the functioning of the amygdala, in elucidating the mechanism of depression. Interestingly, a recent critical quantitative meta-analysis of neuroimaging on the amygdalar processing of emotion indicates that the human amygdala is not only capable of responding to positive as well as negative inputs, but contrary to widely held view, the mean effect size for amygdalar activation by positive stimuli was significantly greater than for negative ones (Sergerie et al., 2008).

### Cross-modal interaction of multimodal stimulation can have synergistic effects on mood regulation

Evidence cited in preceding sections indicates that unisensory stimulation or physical exercise can singly modulate mood and depression. In real life, however, organisms rarely encounter such unimodal inputs but almost always face multimodal stimulation. The multimodal nature of sensorimotor activation in many experimental studies using stress (including the chronic mild stress model of anhedonia; Willner et al., 1992) or enriched environments (Brenes Saenz et al., 2006; Cui et al., 2006) to modulate mood attests to the compound effects of sensorimotor inputs in such treatments. Thus, it will be more realistic to investigate the impact of multichannel rather than unimodal inputs on mood regulation. In light of the ameliorative effects of unimodal stimulation on mood and depressive symptoms, future research may provide more efficacious sensorimotor treatment by systematic investigation of potential synergism by means of multimodal stimulation. There is growing evidence for cross-modal synergism when stimulation from one modality is combined with that from another. After the seminal work by McGurk and MacDonald, 1976) that showed a cross-modal interaction between auditory and visual processing, cross-modal modulation and synergism have been reported across a number of sensory combinations such as between vision and audition (McDonald and Ward, 2000; Watanabe and Shimojo, 2001; Kayser et al., 2009), vision and olfaction (Zellner and Kautz, 1990; Zellner and Whitten, 1999; Koza et al., 2005; Dematte et al., 2009), vision and touch (Kennett et al., 2001; Ro et al., 2004; Gori et al., 2011), olfaction and audition (La Buisonniere-Ariza et al., 2012), somatosensation and audition, as well as audition and gustation (North, 2012).

### Sensorimotor stimulation can be used as an adjunctive treatment in depression

A major aim of research on mood regulation is to provide better means of treating mood disorders. While considerable advances have been made in understanding and treating such disorders, particularly depression, full therapeutic success is not within reach. Monotherapies with a single antidepressant fail to achieve remission in a majority of cases (Entsuah et al., 2001; Trivedi et al., 2006b). As a consequence, a wide net has been cast by both clinicians and researchers in search of more efficient therapies of mood disorders. To this end, new pharmacotherapeutic agents and approaches are being developed (Nestler et al., 2002; Krishnan and Nestler, 2010; Murrough and Charney, 2012; Sanacora et al., 2012) and a range of new therapeutic techniques involving transmagnetic stimulation (Sackeim, 2000; O’Reardon et al., 2007; Andrade et al., 2010), deep brain stimulation (Mayberg et al., 2005), and vagal stimulation (George et al., 2002) are now utilized.

Another approach to overcome the failure of monotherapies in treatment-resistant depression has been to use adjunctive or augmentation treatment in the form of additional pharmacological agents such as lithium or buspirone (Nelson, 2000; Fava, 2001). However, such strategies also have disadvantages such as additional side effects, potential harmful drug interactions as well as added treatment cost. An alternative to co-administration of pharmacological agents is to use sensorimotor stimulation as adjunctive or augmentation therapy. There is already evidence that combining bright-light exposure with exercise ameliorates mood (Partonen and Lönnqvist, 2000; Leppamaki et al., 2002, 2004). Several studies indicate that due to its demonstrated ameliorative effect as monotherapy (Babyak et al., 2000; Blumenthal et al., 2007) exercise can be used adjunctively with pharmacotherapy in treatment of depression (Trivedi et al., 2006a).

### Back to the future: A psychophysical approach to sensorimotor stimulation may provide new insights into mood regulation

An important issue raised by the present review has to do with the fact that it is no longer possible to understand both the top-down and the bottom-up approaches to mood regulation only in terms of the “intensity” or the “valuation” of cognitive and/or viscero-sensory stimulation impinging on an organism. This is because there is now adequate evidence to show that not only excessive but even total absence of sensorimotor input can modulate mood. As shown in previous sections, a deficiency in photic reception or reduced auditory stimulation can cause depressive symptoms in humans and animals. All these point to a very complex central accumulative system that is perturbed by deficient or excessive stimulation which when repeated can cause major mood dysregulations.

An analogy between mood regulatory effects of sensorimotor stimulation and psychophysics is not spurious at this stage for historical as well as neuroscientific reasons. Historically (and conceptually) Fechner’s quest in his pioneering work on psychophysics was to bridge the gap between the physical world impinging on the organism’s sensory systems and the psychological phenomena arising thereof [Fechner, 1987a,b (in translation); see also Scheerer, 1987]. Stevens brought a more valid depiction of the psychophysical law by proposing the power law to replace the Fechnerian logarithmic law. Perhaps just as importantly, Stevens emphasized the neural mechanisms that transduced/translated the sensory information into central physiological activation. Particularly relevant to the present review is Stevens’ research with cross-modal matching whereby the psychological impact of sensory stimulation in one domain/modality is matched with that engendered by stimulation in another (Stevens, 1970). Numerous demonstrations of reliable cross-modal matching in experiments utilizing a large number of dimensional permutations suggest a common coin that allows for a precise and replicable matching of the psychological impacts of cross-modal stimulation. Recent studies employing neuroimaging (Nakashita et al., 2008) and electrophysiological (Giard and Peronnet, 1999; Wang et al., 2008) methods demonstrate neural interactions that may provide the underlying mechanism for such cross-modal neural interactions. Furthermore, two critical structures in the neurocircuitry of depression, namely the amygdala and the prefrontal cortex, are especially wired to co-process inputs from several modalities (Nahm et al., 1993; Cooper et al., 1994; Gilbert et al., 1996; Day et al., 2005; Ghashghaei et al., 2007; Tettamanti et al., 2012). Imaging (Nakashita et al., 2008) and electrophysiological (Giard and Peronnet, 1999; Wang et al., 2008) studies demonstrate neural interactions that may provide the underlying mechanism for cross-modal neural interactions. Related studies have shown that primary sensory cortical areas respond not just to the appropriate unimodal but also to multimodal stimuli. The visual cortex, for instance, can be activated by tactile and auditory stimulation (Hagen et al., 2002; Blake et al., 2004; Beauchamp et al., 2007; Saenz et al., 2008). Similarly, the somatosensory cortex can be activated by visual input (Taylor-Clarke et al., 2002), while auditory cortical neurons respond to somatosensory input (Fu et al., 2003).

In light of these findings, the bottom-up approach to mood regulation points to a common mechanism that accumulates the impact of both ameliorative and detrimental effects of stimulation on mood. Particularly, use of animal models in parametric investigations of the central effects induced by mood regulatory uni-and multimodal stimulation can provide valuable insights into the etiology and treatment of mood disorders, by elucidating those parameters that result in optimal stimulation for psychological well-being.

## Conflict of Interest Statement

The authors declare that the research was conducted in the absence of any commercial or financial relationships that could be construed as a potential conflict of interest.
